# Use of computed tomography coronary calcium score for prediction of cardiovascular events in cancer patients: a retrospective cohort analysis

**DOI:** 10.1186/s40959-023-00196-9

**Published:** 2024-01-02

**Authors:** Sinal Patel, Francisco X. Franco, Malcolm McDonald, Carlos Rivera, Bernardo Perez-Villa, Patrick Collier, Rohit Moudgil, Neha Gupta, Diego B. Sadler

**Affiliations:** 1 Cleveland Clinic Lerner College of Medicine, Case Western Reserve University, Cleveland, OH USA; 2 Cardio Oncology, Robert and Suzanne Tomsich Department of Cardiovascular Medicine. Heart, Vascular and Thoracic Institute, Cleveland Clinic Florida, 2950 Cleveland Clinic Blvd, Weston, FL 33331 USA

## Abstract

**Background:**

CT- coronary calcium score, is one of the most studied and widely available modalities in cardiovascular medicine. Coronary artery calcium score (CACS) is an established predictor of coronary artery disease. The ‘standard of care’ diagnostic modality to measure CACS is ECG-gated Cardiac Multi-Detector Computed Tomography. There is convincing evidence of a strong association between CACS and major cardiovascular (CV) events in asymptomatic individuals. Cancer patients (C) may have a higher risk for CV disease than non-cancer patients (NC) related not only to cancer treatments but also to shared biological factors and pathways. Thus, identifying tools for early detection of CV disease in this population is of utmost importance.

**Methods:**

A retrospective cohort analysis was performed with patients from Cleveland Clinic Florida and Ohio who had CACS from 2017 to 2021. Patients who had cancer diagnosis prior to CACS were matched to NC for age and sex. CV events after their index CACS events were compared between C and NC, and matched control and propensity analysis were conducted.

**Results:**

Ten thousand seven hundred forty-two patients had CACS; 703 cancer patients had CACS and were eligible. Extensive CACS (> 400) were significantly higher in cancer, 94 (13.37%) vs non-cancer patients, 76 (10.83%), *P* = 0.011. Furthermore, after propensity matched analysis, CACS > 400 was 14.8% in C vs 9.6% in NC, *P* =  < 0.05. CV events were similar in both cohorts (*p* = NS), despite less CV risk factors in cancer patients (*P* =  < 0.05). For the combined moderate (101–400) & extensive (> 400) CACS, the prevalence of stroke and peripheral arterial disease, a marker of systemic atherosclerosis, was significantly higher in patients with cancer (*P* < 0.01).

**Conclusions:**

Despite having fewer CV risk factors in our study, similar CACS in cancer patients are suggestive of a higher prevalence of CV disease independent of traditional risk factors. High CACS and the overall prevalence of vascular events were more frequent in patients with cancer. Higher prevalence of peripheral arterial disease and cerebrovascular accident further suggests the increased atherosclerotic burden in C.

## Background

Cardiovascular disease (CVD) is the leading cause of mortality worldwide. Approximately 17.9 million people die from CVD every year, accounting for 31% of all global deaths [1]. Behind CVD, cancer is responsible for the second-most deaths in the global population, with the most recent data attributing over 10 million deaths to cancer annually worldwide [2]. It is increasingly understood that these two disease processes not only pose a mortality threat individually but often behave synergistically to promote an inflammatory environment that hastens mortality and worsens numerous systemic comorbidities [3, 4]. Cancer patients (C) are estimated to, on average, have a 42% greater relative risk for cardiovascular disease and death when compared to non-cancer patients (NC) in the general population [5]. Furthermore, with advances in cancer care and with the introduction of new treatment modalities that prolong patients’ longevity, underlying comorbidities and cancer treatment-related toxicities’ impact on morbidity and mortality are more pertinent than ever [3, 6]. For these reasons, tools for the early detection of developing CVD in cancer patients are needed.

One such stratification tool is the coronary artery calcium score (CACS). Typically performed via ECG-gated cardiac multi-detector computed tomography (MDCT) or derived from routine computed tomography (CT) of the chest, the CACS is one of the most thoroughly studied and widely available tests in cardiovascular medicine [7–9]. Multiple large, long-term, population-based observational studies out of the United States, Germany, and the Netherlands have built a large body of evidence demonstrating a strong association between CACS and major cardiovascular outcomes in asymptomatic patients [10–13]. As a result, current clinical practice guidelines in the United States and Europe recommend CACS as a useful way to predict the risk of subclinical cardiovascular disease (CVD) in asymptomatic patients, allowing for risk stratification and guidance of follow-up testing and management [14–17]. Thus, low CACS can limit the need for follow-up examinations, unnecessary testing, and excessive intervention while, conversely, a high or increasing CACS can presage an increasing risk for cardiovascular events and the need for more aggressive preventive measures.

Despite the firm establishment of CACS as a powerful risk stratification tool in the general population, specific recommendations regarding its application to the cancer patient population have not yet been developed. The physiologic effects of malignancy extend far beyond a focal tumor. It is proposed that through mechanisms such as pro-inflammatory cytokine release, increased oxidative stress, and promotion of a pro-thrombotic state, cancer can independently alter the body’s chemistry on a systemic scale [18]. These mechanisms, coupled with the noxious chemo-, radio-, immuno- and hormonal therapies that cancer patients are regularly exposed to, are consistently demonstrated to result in a statistically higher burden of CVD with earlier onset of clinically significant disease on average [19, 20]. Moreover, increasingly used immune checkpoint inhibitors, while highly effective, have been shown to have a direct role in development of accelerated atherosclerosis [20–22].

This study sought to evaluate the burden of atherosclerosis and CV events on patients with cancer using the CACS as a screening tool to detect CVD most effectively in this vulnerable population. We examined the association of CACS with CVD events in C vs. NC.

## Methods

A retrospective cohort analysis from Cleveland Clinic Florida and Cleveland Clinic Ohio was performed on patients from 2017 to 2021 (5 years). Patients who had CACS during that period were identified and cancer patients (C) who had a CT CACS after the cancer diagnosis were selected (C) and compared to an age- and a sex-matched cohort of non-cancer patients (NC).

CACS reported by staff radiologists at the time of test performance were utilized for analysis.

The Student T-test of unequal variances was used for continuous variables and Chi-square for categorical variables.

Five cardiovascular events that occurred after CACS date were individually evaluated by retrospective chart review of the electronic medical record: coronary artery disease, heart failure, myocardial infarction, cerebrovascular accident (CVA) and peripheral arterial disease (PAD). Events were adjudicated from the electronic records diagnoses.

Primary goals were to compare the CV risk factors and CACS in C vs. NC patients and the prevalence of CVD events in these two groups according to their CACS.

**Fig. 1:**
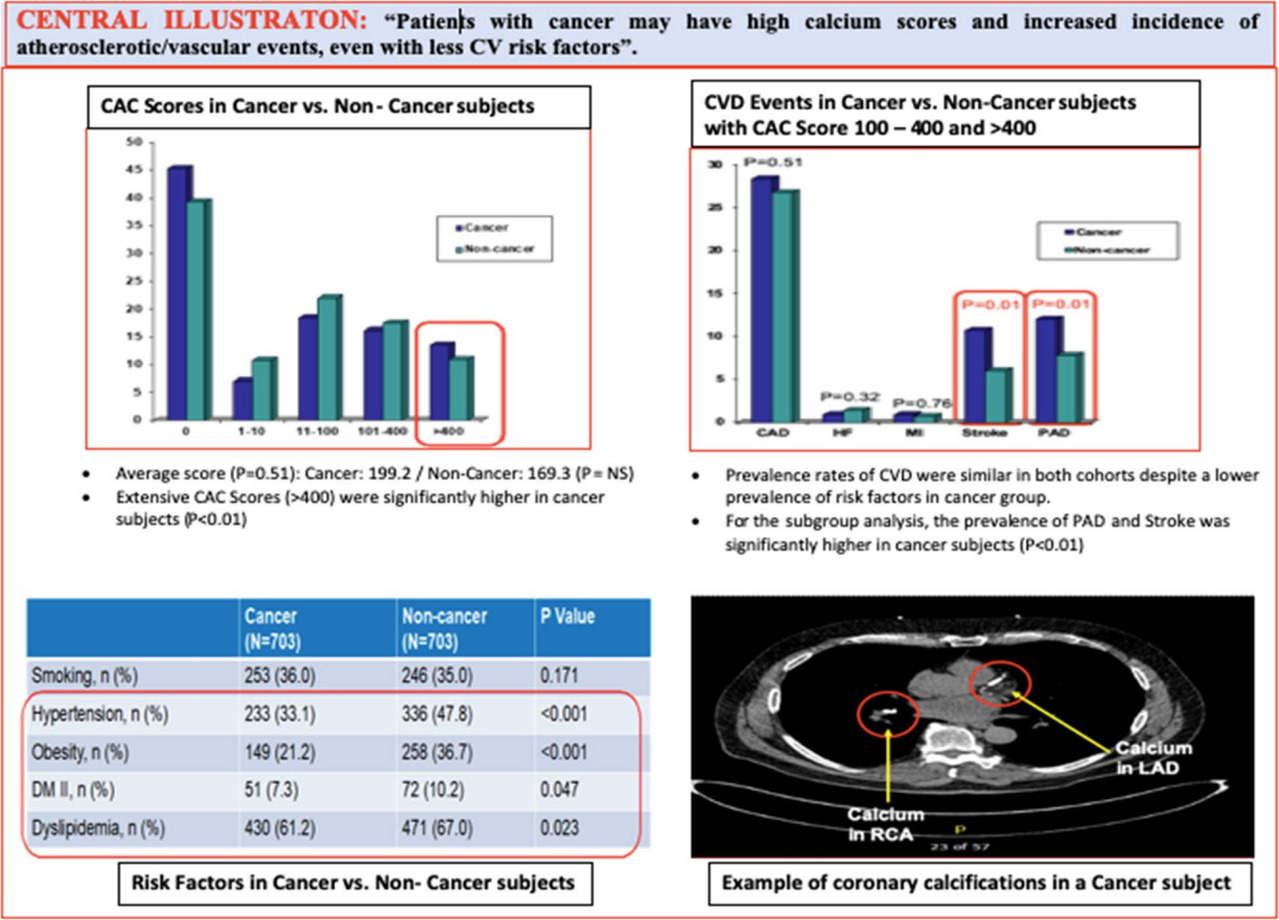
Central Illustration: Patients with C may have high CACS and increased incidence of atherosclerotic/vascular events, even when having less CV risk factors

Propensity-score matching was performed by using the nearest neighbor method with a caliper of 0.05 standard deviation of the logit. Variables utilized for propensity matching included hypertension, obesity, diabetes, dyslipidemia, and history of cigarette smoking. A standardized mean difference < 0.1 (10%) was used for each of the matching variables. A subgroup analysis was performed to evaluate any differences in primary endpoint in patients with CACS with or without cancer. Univariate analysis was conducted to compare and summarize characteristics and outcomes between groups. A multivariate regression model was performed to assess the differences between groups and adjust for unbalanced characteristics. We established a statistical significance level of 0.05 Fig. 1.

## Results

**Fig. 2:**
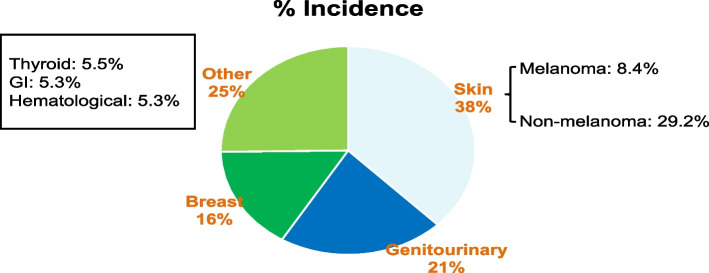
Prevalence of different C types in CACS patients' population

A total of 10,742 patients had CACS during the study period. 1016 patients had cancer. From that group, 703 had complete data at the Cleveland Clinic electronic health care records (EHR) and were the subject group of this study: they were analyzed (C) and compared to 703 NC. Mean age was 61.4 ± 8.6 (C) and 61.3 ± 8.5 years (NC). Melanoma and nonmelanoma skin (37.6%), genitourinary (21.1%), and breast (16.1%) were the most prevalent cancer types (Fig. 2). Mean CACS was 199.2 in C and 169.3 in NC (*P* = 0.51). CACS were higher in C, in the > 400 category, 94 (13.37%) vs 76 in NC (10.83%), (*p* = 0.011). The prevalence of CVD events was similar in both cohorts despite a lower prevalence of hypertension, obesity, diabetes, and dyslipidemia in C  (Table 1). CVA (*p* = 0.001) and PAD (*p* = 0.010) were more frequent in C than NC (Table 1). For the subgroup with the combined 101–400 and > 400 CACS, C had a higher incidence of PAD, a marker of systemic atherosclerosis, *p* = 0.009 (Central Illustration).

**Table 1:**
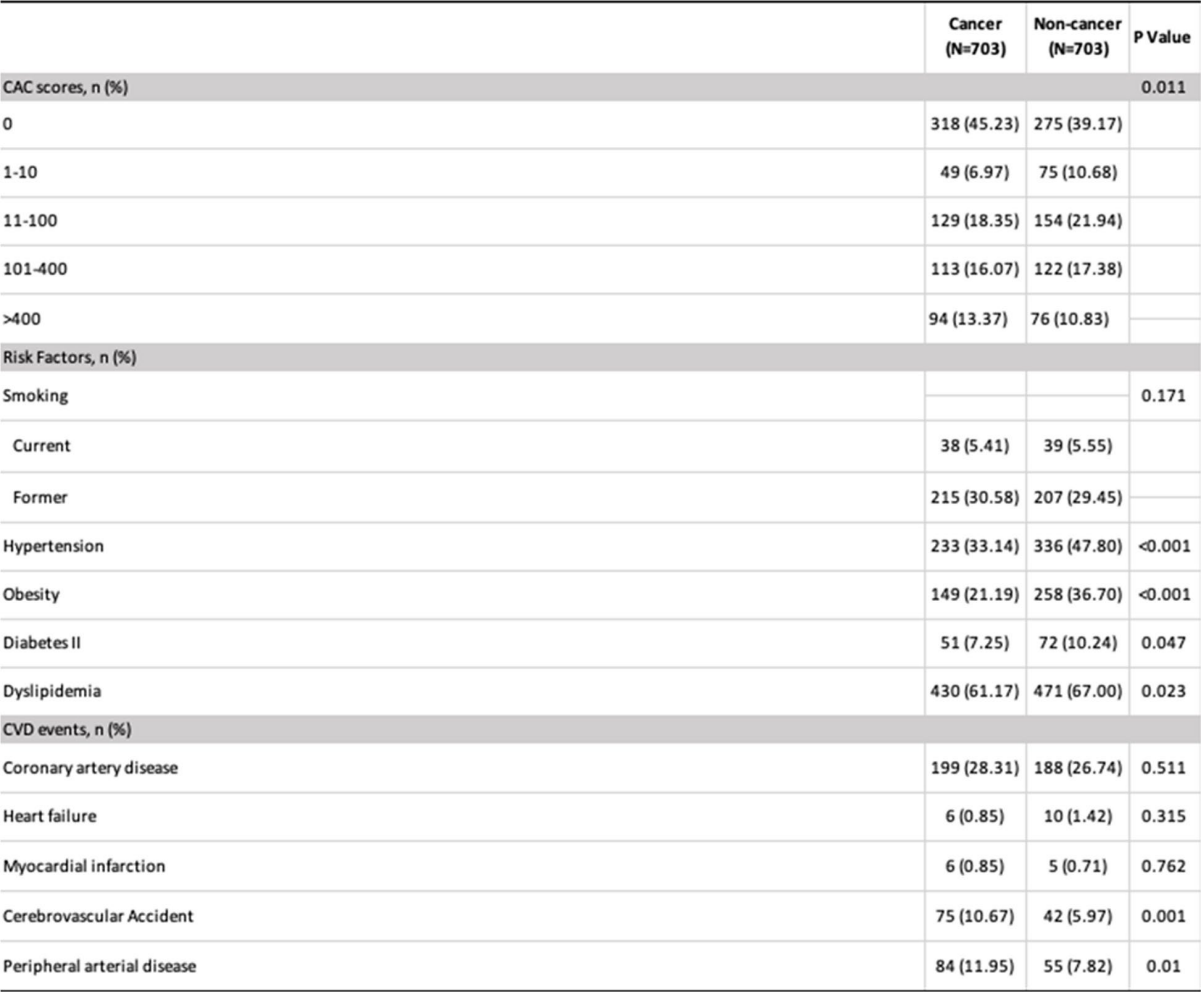
The prevalence of CVD events was similar in both cohorts despite a lower prevalence of CV risk factors in C subjects

*Abbreviations: CAC CT* coronary artery calcium, *CVD* cardiovascular disease

A summary of the means and percentages of the baseline variables for each group can be found in Table 1. In this cohort of patients who underwent CACS, C had a lower prevalence of hypertension (*P* =  < 0.001), obesity (*P* =  < 0.001), Diabetes (*P* = 0.047) and dyslipidemia (*P* = 0.023) compared to NC. It should be noticed that, even without correction by CV risk factors, there was a higher prevalence of CVA: 75 (10.67%) vs 42 (5.97%), *P* = 0.001 and PAD: 84 (11.95%) vs 55 (7.82%), *P* = 0.01 in C vs NC (Table 1 and Central Illustration).

Propensity scores matched analysis correcting by CV risk factors was conducted to minimize potential confounding results from the primary matched controlled analysis. A summary of the means and percentages of the variables for each group can be found in Table 2. In this propensity analysis, after correcting for CV risk factors, lower CACS (0, 1–10, and 11–100) were slightly higher in NC than in C: 428 (74.69%) vs 397 (69.28%), *P* = NS. There were no differences between both groups for the intermediate CACS 101–400: NC 89 (15.6%) vs C 91 (15.9%). However, the highest CACS > 400 was more frequent among C, 85 (14.8%) vs NC, 55 (9.4%), *P* =  < 0.01. After adjustment by propensity scores, there was a higher incidence of CVA, PAD and in this analysis also CAD in C.

**Table 2:**
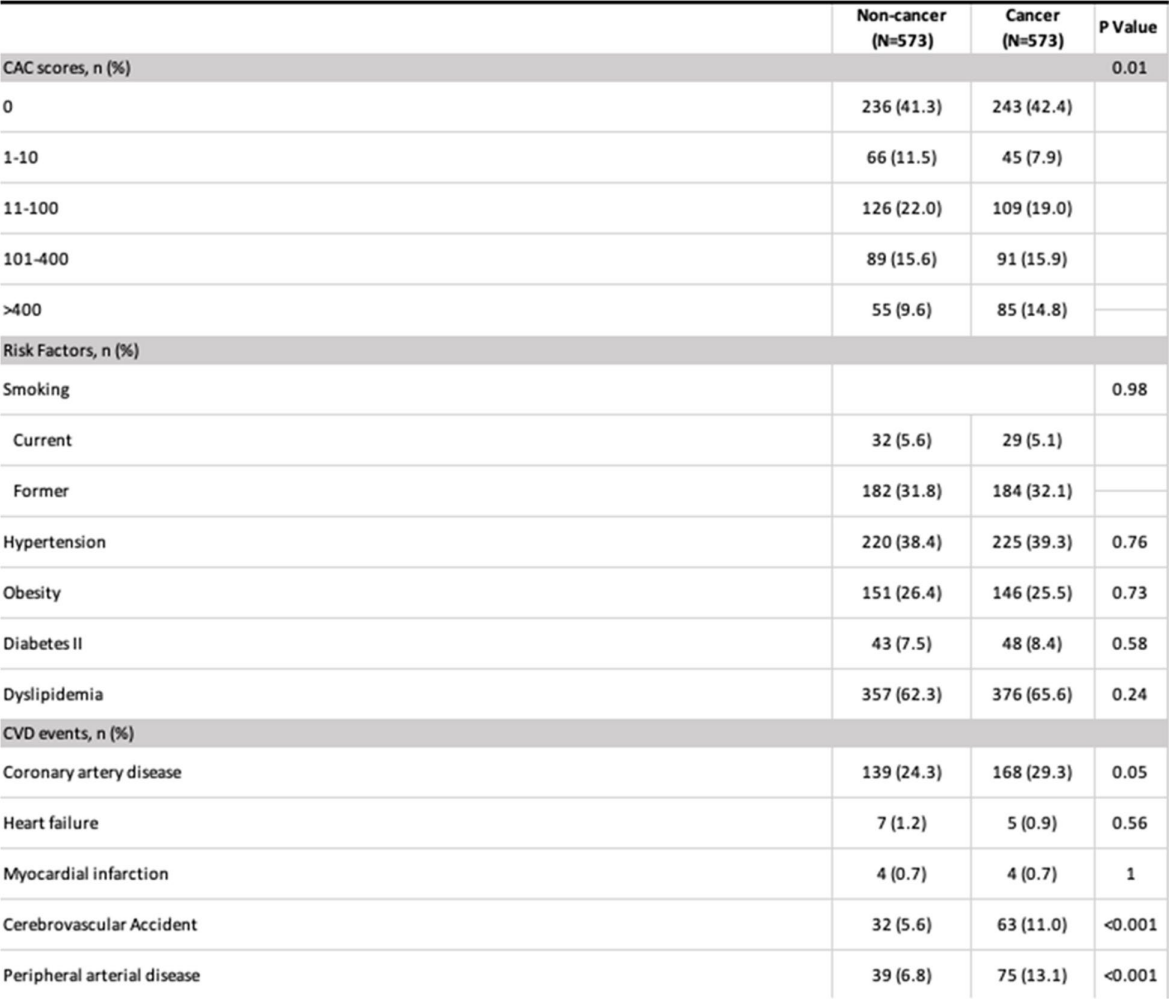
Calcium score corrected by propensity matched analysis for patients with CACS > 400: It was significantly higher for C vs NC. PAD, Stroke and CAD were more prevalent in C vs NC for this subgroup

*Abbreviations: CAC CT* coronary artery calcium, *CVD* cardiovascular disease

## Discussion

This study sought to assess the CACS as a targeted cardiovascular risk stratification tool in an at-risk population. Though CACS is a well-established screening tool in the general population [7, 23, 24], in this retrospective analysis of over 700 cancer patients, we observed that cancer patients showed significant elevations in levels of coronary calcium scores despite having a lower prevalence of traditional CV risk factors, and when cross-matched with non-cancer counterparts also exhibited an excess of atherosclerotic cardiovascular disease (ASCVD) by quantifiable calcific burden (Table 1). With statistically lower rates of common CVD risk factors including hypertension, obesity, diabetes mellitus type 2, and dyslipidemia, cancer patients were found to have equivalent rates of CVD events (defined as coronary artery disease, heart failure, myocardial infarction, cerebrovascular accident, or peripheral arterial disease). This highlights the possibility and likelihood of cancer diagnosis and treatment being independent risk factors in the development of CVD and suggests that clinical risk stratification models centered on common risk factor profiles and clinical characteristics may underestimate risk in this population.

Furthermore, cancer patients with moderate (101–400) and extensive (> 400) CACS were found to have significantly increased prevalenceof atherosclerotic calcific burden, including both PAD and CVA, in comparison to their non-cancer counterparts (Table 2). This is in keeping with prior studies exemplified by Whitlock, et al. [24], which showed the association between cancer diagnosis and treatment with the development of coronary calcifications. Recognizing the incremental value of these findings is critical. Our findings, in building on those of prior studies, suggest that CACS may serve as a risk stratification tool for the identification of an at-risk for CV disease cancer patients’ population. Early identification of CV risk may lead to implementation of preventive therapies.

The proposed mechanisms underlying the link between cancer and CVD center primarily on the role of cancer in promoting local and systemic inflammatory cascades. Inflammasomes, interleukins, and pro-inflammatory cytokines that are commonly elevated in cancer patients have been linked to accelerated atherogenesis [25–28]. Furthermore, proposed secondary mechanisms such as biomarker-mediated alterations in metabolism [13, 29] suggest that mitigation of risk through dietary, lifestyle, and risk factors modification alone is likely insufficient.

Beyond the direct pathophysiological impacts of cancer itself, the effects of the many therapies used to treat cancer cannot be ignored. Chemotherapeutic agents such as tyrosine kinase inhibitors, platinum agents, taxanes, 5-fluorouricil, and hormonal therapies have all been implicated in the development of CVD through mechanisms such as direct endothelial cell injury, oxidative stress, myocardial ischemia through vasospasm, arterial thrombosis, and promoting CVD risk factors such as relevant systemic hypertension [3, 30–33]. These impacts are only further compounded by exposures to other noxious therapies such as radiation therapy and androgen deprivation therapy, both commonly implicated in the development of CVD [18, 34–36].

Therapies well-established for ASCVD risk reduction in the general population are not without risk, especially in cancer patients who are prone to several iatrogenic and intrinsic comorbidities such as myelosuppression and end-organ failure [37, 38]. Therefore, risk stratification tools in this population must not only assess for the presence of ASCVD, but also help guiding the need for safe and early intervention [39].

Though beyond the scope of this study, our data on increased incidence of atherosclerotic disease by relative calcific burden may also suggest the need for future establishment of adjusted CACS thresholds that are specific to this at-risk population and highlights a need for further dedicated investigations. Most prior studies on this topic have been limited to small, cross-sectional cohorts, often focused on one specific malignancy. To our knowledge, our cohort of over 700 patients, representing a wide variety of cancer types, is one of the largest of its kind.

### Study limitations

Potential for referral bias could exist, but this retrospective analysis is not equipped to evaluate for this bias.

Like previous investigations into this topic, limitations of this study include the lack of data regarding the specific therapy received by each patient undergoing cancer treatment, representing a possible confounding contributor to outcomes and CVD burden. This highlights a need for a dedicated study into CACS profiles and risk stratification according to the type of therapy received. Furthermore, the single-center and retrospective nature of the analysis limits the statistical power of the study, precluding stratification by individual cancer type to better determine specific screening and intervention needs by type of malignancy. Randomized, prospective investigation into the topic will be required.

Short-term follow-up was deemed a limitation in correlating CACS with CVD events such as myocardial infarction and heart failure. Future analyses are needed with a focus on CACS profiles by malignancy sub-type and treatment modality to better assess the CVD impact on each individual scenario. Considering the increasingly understood risk of CVD faced by cancer patients across all sub-types, studies like these may yield new applications of widely available CACS to improve detection, stratification, and intervention of CVD in this vulnerable population.

## Conclusions

Comparing an age- & sex-matched cohort of patients with cancer vs. non-cancer, we found similar CACS scores and prevalence of CVD despite a lower relative burden of CV risk factors. Ranges of CACS scoring and the prevalence of CVD events in cancer patients were no different from non-cancer group, suggesting higher prevalence after adjusting for risk factors. A propensity analysis was made to confirm this finding. Cancer patients with high CACS scores had a higher incidence of PAD. Given the retrospective single-center nature of this study, we propose additional prospective multicenter studies to better assess CACS in patients with cancer.

We acknowledge that this comparison is limited by the database, hence further prospective studies are needed to confirm, expand, and better understand the mechanisms behind our results.

### Clinical perspective

Patients with cancer may have high calcium scores and increased risk of atherosclerotic /vascular events, even when they have less CV risk factors, suggesting an independent increased risk of atherosclerosis/CVD present in the cancer population.

## Data Availability

The datasets used and/or analyzed during the current study are available from the corresponding author on reasonable request.

## Abbreviations

CACS: Coronary artery calcium score
CV: Cardiovascular
CT: Computed tomography
CAD: Coronary artery disease
MDCT: Multi-detector computed tomography
HER: Electronic health care records
C: Cancer patients
NC: Non-cancer patients
CVD: Cardiovascular disease
PAD: Peripheral arterial disease
CVA: Cerebrovascular accident
ASCVD: Atherosclerotic cardiovascular disease
